# Disturbed Matrix Metalloproteinase Pathway in Both Age-Related Macular Degeneration and Alzheimer's Disease 

**DOI:** 10.1155/2017/4810232

**Published:** 2017-01-18

**Authors:** Ali Aijaz Hussain, Yunhee Lee, Jin-Jun Zhang, Paul T. Francis, John Marshall

**Affiliations:** ^1^Department of Genetics, UCL Institute of Ophthalmology, London, UK; ^2^Nanobiotech Co., Ltd., Heungdeok IT Valley, Yongin, Republic of Korea; ^3^Wolfson Centre for Age-Related Diseases, King's College London, London, UK

## Abstract

*Purpose.* Abnormal protein deposits including *β*-amyloid, found in ageing Bruch's membrane and brain, are susceptible to degradation by matrix metalloproteinases (MMPs). In ageing Bruch's membrane, these MMPs become less effective due to polymerisation and aggregation reactions (constituting the MMP Pathway), a situation much advanced in age-related macular degeneration (AMD). The likely presence of this MMP Pathway in brain with the potential to compromise the degradation of *β*-amyloid associated with Alzheimer's disease (AD) has been investigated.* Methods.* Presence of high molecular weight MMP species (HMW1 and HMW2) together with the much larger aggregate termed LMMC was determined by standard zymographic techniques. Centrigugation and gel filtration techniques were used to separate and quantify the distribution between bound and free MMP species.* Results.* The MMP Pathway, initially identified in Bruch's membrane, was also present in brain tissue. The various MMP species displayed bound-free equilibrium and in AD samples, the amount of bound HMW1 and pro-MMP9 species was significantly reduced (*p* < 0.05). The abnormal operation of the MMP Pathway in AD served to reduce the degradation potential of the MMP system.* Conclusion.* The presence and abnormalities of the MMP Pathway in both brain and ocular tissues may therefore contribute to the anomalous deposits associated with AD and AMD.

## 1. Introduction

Alzheimer's disease (AD) and age-related macular degeneration (AMD) are neurodegenerative disorders that share a strong correlation with advancing age and are both characterized by similar extracellular deposits. Common constituents within the deposits include vitronectin, apolipoprotein E, amyloid P, amyloid *β* (A*β*), lipids, inflammatory mediators, and complement components [1–4]. Whereas AD shows the abundant presence of aggregated A*β* peptides and hyperphosphorylated tau protein in brain, AMD is characterized by deposition of lipids, lipoproteinaceous debris, and oxidized and damaged extracellular matrix (ECM) components within Bruch's membrane of the eye [5–7].

Deposits accumulate when there is an imbalance between production and clearance. In Bruch's membrane, tightly coupled processes of synthesis and degradation serve to continuously turn over the ECM of the membrane, thereby maintaining its structural and functional characteristics. The degradation pathway is mediated by matrix metalloproteinases (MMPs) and primary gelatinase components are MMP2 and MMP9, the former being the constitutive enzyme and the latter the inducible form. These MMPs are released as inactive proenzymes (pro-MMPs) and on activation (by proteolytic cleavage of a small peptide) are capable of digesting all components of an ECM [8–11]. MMPs are involved in many diverse tissue remodelling activities including wound healing and embryonic development, and increased activities are associated with pathological conditions as varied as cancer, rheumatoid arthritis, and cardiovascular diseases [12–16].

In normal ageing, the increased thickness of Bruch's membrane [17, 18], the deposition of normal and abnormal ECM material [6], increased cross-link formation (oxidative and nonenzymatic glycosylation leading to advanced glycation end-products, AGEs, and ALEs) [19], and the accumulation of lipid-rich debris [20, 21] are changes that implicate a disturbance in the ECM turnover of the membrane. Irrespective of an age-related increase in levels of pro-MMP2 and pro-MMP9 in Bruch's membrane, the amount of oxidized, denatured, and damaged collagenous substrate was observed to increase, accounting for nearly 50% of total collagen in elderly membranes [6, 22]. In advanced ageing associated with AMD, despite a 2-fold increase in the total level of pro-MMP9, levels of active-MMP2 and active-MMP9 were reduced by 50% compared to age-matched controls [23]. Thus diminished degradative capacity appears to shift the balance towards accumulation of normal and abnormal constituents.

**Figure 1:**
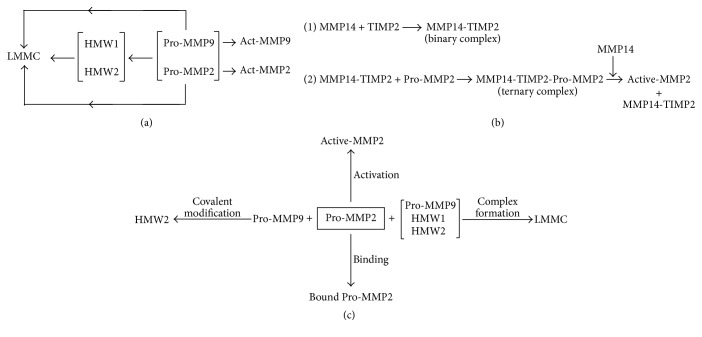
(a) The Matrix Metalloproteinase (MMP) Pathway. The gelatinase components comprise the pro- and active forms of the monomeric MMP2 and MMP9, the high molecular weight forms denoted by HMW1 and HMW2, and the large macromolecular weight MMP complex termed LMMC consisting of HMW1 and HMW2, pro-MMP9, and a trace of pro-MMP2. Apart from LMMC, all other gelatinase components have been shown to exist in a free/bound equilibrium (pathway constructed from reference [38].) (b) Activation scheme for pro-MMP2. (c) Competing reactions for free pro-MMP2. These are primarily binding, activation, covalent interaction with pro-MMP9 to form the HMW2 species, and incorporation into the LMMC complex (constructed from [24–27, 38, 39]).

Age-related mechanisms in Bruch's membrane that result in increased levels of pro-MMPs but decreased levels of activated enzymes are poorly understood. The MMP Pathway (Figure 1(a)) shows the myriad of reactions undergone by free pro-MMPs to produce the high molecular weight species designated HMW1, HMW2, and the large macromolecular weight MMP complex (LMMC). A further complication is that all these species exist in a free and bound equilibrium and therefore the operation of the MMP Pathway effectively sequesters-free pro-MMPs. The likely impact of these competitive reactions on the activation of the constitutive MMP2 enzyme is illustrated in Figures 1(b) and 1(c).

Pro-MMP2 activation is mediated by another metalloproteinase, a transmembrane enzyme MMP14, in combination with a tissue inhibitor of MMPs and TIMP2 [24–26]. The activation requires two molecules of MMP14; the first MMP14 molecule binds TIMP2 and this enables the formation of the ternary complex with pro-MMP2. A second MMP14 molecule then cleaves the proform to release active-MMP2 [27]. Thus, efficient activation requires the presence of MMPs and TIMPs in optimum concentrations. Competing reactions that reduce the level of free pro-MMP2 are the covalent interaction with pro-MMP9 to form HMW2, binding within the extracellular matrix, and incorporation of pro-MMP2 into the LMMC complex. In AMD, the formation of HMW2 is enhanced by the >3-fold increase in the free level of pro-MMP9 resulting in decreased levels of the active-MMP2 enzyme [23].

In brain, A*β* is formed by the sequential cleavage of the membrane-bound amyloid beta precursor protein (A*β*PP) by *β*-secretase (BACE1) and the presenilin-dependent *γ*-secretase. Proteolysis by *γ*-secretase leads to a variety of A*β* species, the major portion (~80–90%) being of 40 amino acid residues (A*β*40) followed by a 42-amino acid peptide (5–10%, A*β*42), the latter being more hydrophobic and constituting the main deposit in the brain [28]. Similar pathways are also known to be present in the retinal pigment epithelium (RPE) accounting for the presence of A*β* in Bruch's membrane [29, 30].

Removal of A*β* occurs via transport mechanisms and enzymatic hydrolysis. Transport is by drainage along perivascular basement membranes and receptor mediated translocation across the blood-brain barrier (BBB) [31, 32]. The A*β* degrading enzymes (ADEs) comprise over 20 multifunctional metalloendopeptidases and include neprilysin (NEP), insulin-degrading enzyme (IDE), angiotensin-converting enzyme (ACE), endothelin-converting enzyme (ECE), and MMPs [33–36]. Of these various enzyme systems, only MMP9 was able to degrade fibrillar A*β* (fA*β*) in vitro and compact plaques in situ [37].

Measurement of enzymatic activities in postmortem brains showed that NEP, IDE, ACE, ECE-1, ECE-2, and MMPs were all higher in AD [37, 40–44]. These studies are perhaps verified by cell culture analyses that have shown upregulation of ADEs such as NEP, ECE, ACE, ECE-2, IDE, and MMP2, MMP3, and MMP9 on exposure to A*β* [42, 43, 45–47].

Thus in AD, despite the elevated presence of ADEs, the abnormal A*β* product continues to accumulate. This anomalous situation (of high levels of latent enzymes and reduced substrate degradation) is similar to that observed in normal and abnormal ageing of Bruch's membrane. It is therefore highly likely that MMP transformations akin to the MMP Pathway in Bruch's membrane (Figure 1(a)) may also operate in brain tissue leading to sequestration of free MMP species, curtailing their activation potential. There is some evidence for the presence of high molecular weight MMP species (HMW1 and HMW2) in both human AD brain and in animal models of the disease [48, 49].

In this preliminary investigation, we have demonstrated the presence of the MMP Pathway in brain tissue and assessed its operation in donors with AD.

## 2. Materials and Methods

### 2.1. Tissue Preparation

After securing Research Ethics Approval, brain donor samples from the parietal cortex (~0.5–1 g/donor) were obtained from Brains for Dementia Research Unit/Bristol Brain Bank of Bristol University, UK. Parietal cortex tissue was chosen because it was readily available in sufficient quantity to optimise the analytical procedures described in this study. Tissue samples were transported to the laboratory on dry ice and stored at −70°C until experimentation. Altogether, 4 control samples (age 81 ± 5 years; postmortem delay 37.5 ± 7 hours; Braak stage 1.8 ± 0.4) and 5 AD affected samples (age 79 ± 4; postmortem delay 37 ± 21 hours; Braak stage 5.4 ± 0.5) were made available for these studies (mean ± SD, Table 1).

Brain samples were processed to obtain three tissue fractions: homogenates, supernatants, and insoluble tissue pellets. About 50–100 mg wet weight of sample was homogenized in 1.0 mL Tris-buffered saline (TBS, 50 mM Tris, 0.15 M NaCl, 10 mM CaCl_2_, and 0.02% sodium azide, pH 7.4) using 20 strokes of a hand held 3.0 mL Potter-Elvehjem homogenizer (clearance 0.004–0.006 in, Sigma-Aldrich, UK). After withdrawal of a 50 *μ*L aliquot for protein estimation by the Folin-Ciocalteu method [50], some of the homogenates were spun at 10,000*g* for 5 minutes to obtain the supernatant and pellet fractions. Supernatant and pellet proteins were also measured.

Bruch's membrane was prepared from the eye of a donor aged 76 years, obtained from the Bristol Eye Bank, UK, as previously described [23]. Briefly, a circumferential incision was made 5 mm posterior to the sclera sulcus and the anterior segment discarded. The remaining globe was opened in the shape of a Maltese cross and the four segments separated and transferred to a Petri dish containing TBS. Using a pair of fine forceps, the retina and vitreous were carefully removed. The exposed monolayer of retinal pigment epithelial (RPE) cells was brushed away with a camel's hair brush exposing Bruch's membrane. After transferring the preparation to fresh TBS, an 8 mm trephine was used to remove a full thickness button of Bruch's membrane to sclera. Finally, the Bruch's-choroid sample was removed by blunt dissection from the underlying sclera.

### 2.2. Identification of MMP Species Except LMMC

Homogenates were screened for the presence of MMP species by substrate gelatin zymography [22, 23, 38, 39]. Zymography involves the electrophoretic separation of MMPs according to molecular weight, and their identification following degradation of the substrate incorporated into the gel. It is a relatively simple, quantifiable, and functional assay for MMPs [51–53]. Briefly, proteins are separated in a polyacrylamide gel containing a MMP substrate (such as gelatin) and the presence of sodium dodecyl sulfate (SDS) in the gel leads to denaturation of MMPs [54]. After electrophoresis, the gel is incubated in Triton X-100 which exchanges for SDS leading to partial activation of the MMPs [55–57]. Gels are then incubated in activation buffer allowing the MMPs to digest the substrate [52, 58]. Following staining of the gel with Coomassie blue, the MMP containing regions are observed as transparent (or clear) bands against a blue background. These bands are then quantified by densitometry [56, 59].

The level of individual MMP species in both homogenates and supernatants was quantified to obtain the amount of a given species in the bound state.

Because MMPs exist in an equilibrium between bound and free species, tissue homogenization will inevitably lead to dilution and this may alter the amount bound and the extent of this redistribution will be dependent on the magnitude of the dissociation constants. To assess this phenomenon, homogenates were diluted and after a 6 hour incubation, the amount of free MMPs in the supernatants was determined.

Brain tissue (346 mg) from control donor number 2 was homogenized in 4.0 mls Tris buffer and six 0.5 mls aliquots prepared. Three of these were diluted 1/10 by addition of 4.5 mls TBS (termed preparation A) and all tubes incubated at 37°C for 6 hours. After incubation, the samples were centrifuged at 3000 ×g for 20 mins to obtain pellet and supernatant fractions. Supernatants from the 0.5 mls samples were then diluted 1/10 with TBS (termed preparation B). Both preparations were then subjected to zymographic analysis.

Homogenization of the tissue sample results in the release of the LMMC complex into the supernatant. Use of sodium dodecyl sulfate (SDS) for preparing samples for zymography results in dissolution of the LMMC particle into its constituents. Therefore the measured MMP content in the supernatant will include both free MMP species and those released from the LMMC.

### 2.3. Isolation of the LMMC Complex and Determination of Free Levels of MMP Species

LMMC is a large macromolecular weight complex found in the extracellular compartment with its constituents HMW1, HMW2, pro-MMP9, and some pro-MMP2, all held together by noncovalent attachment. It is therefore easily dispersed in SDS containing buffers and thus cannot be examined directly in supernatants by zymographic techniques. Because of its large size, it is excluded from Sepharose CL-6B gel filtration media and exits the column in the void volume with a *v*_*e*_/*v*_*o*_ ratio of about 1.0. Subsequent zymographic identification of its constituents at the forefront of the elution profile is indicative of the presence of the LMMC complex.

The protein content of the supernatant fraction was adjusted to 3.57 mg/0.8 mls and applied to a Sepharose CL-6B filtration column (30 cm × 1.5 cm i.d.) preequilibrated with TBS. Flow rate was adjusted to 0.6 mls per minute and 1.2 mls fractions collected for a period of 1.5 to 2 hours. The protein profile of the chromatography run was obtained by measuring the absorbance of the fractions at a wavelength of 280 nm. Fraction aliquots were then processed for zymographic analysis as described below. The intensity of the bands of individual gelatinases was plotted as a function of eluted volume (*v*_*e*_/*v*_*o*_) to determine their relative distribution between the LMMC species and the free state.

### 2.4. Gelatin Zymography

Samples for zymography were mixed 1 : 1 (v/v) in SDS sample buffer (62.5 mM Tris, 10% glycerol, 4% SDS, and 0.05% bromophenol blue), vortexed, and spun (10,000*g*/5 mins) and 30 uL aliquots applied to gel lanes.

For zymography, 10% SDS-PAGE gels (1.0 mm thick) were prepared containing a 4% stacking layer and 0.1% gelatin in the separating layer. Samples for analysis were loaded into lanes together with prestained protein molecular weight markers (Invitrogen, UK) and 10% foetal calf serum (FCS, Sigma-Aldrich, UK) as an internal standard to correct for gel-to-gel variation in background staining. Electrophoresis was performed using the X Cell SureLock Mini-Cell system (Invitrogen, UK).

After electrophoresis (180 V, 1 hour), the gels were removed from their cassettes, rinsed in distilled water, and incubated for two half-hour periods in 2.5% Triton X-100 to remove SDS and renature the proteins (as described earlier). They were then transferred to reaction buffer (50 mM Tris-HCI, 10 mM CaCl_2_, 75 mM NaCl, and 0.02% NaN_3_, pH 7.4) and incubated at 37°C for 20 hours to allow proteolytic digestion of the gelatin substrate. Gels were rinsed again in distilled water and stained with Coomassie blue (0.1% Coomassie blue R-250, 40% methanol, 10% acetic acid) for 30 minutes. Destaining was carried out for 30 minutes with 40% methanol and 10% acetic acid solution.

MMP activity was observed as transparent bands on a blue background. These gels were scanned at a resolution of 2400 dpi (Epsom 3490 scanner) and stored in JPEG format. The colour images were uploaded into the Quantiscan software package (Quantiscan Version 3.0, Biosoft, Cambridge, UK) in grey-scale format, colours inverted so that MMPs were now visualised as dark bands against a whitish background, and after background correction, the area under the densitometric scan for an individual gelatinase band was quantified.

### 2.5. Statistical Analysis

All data are presented as mean ± SD (*n*) with n being the number of donors. Student's *t*-test was used to assess significance between the amount of free pro-MMP9 in the elution profiles between the control and AD samples.

## 3. Results

### 3.1. Components of the MMP Pathway and Free/Bound Levels

**Figure 2:**
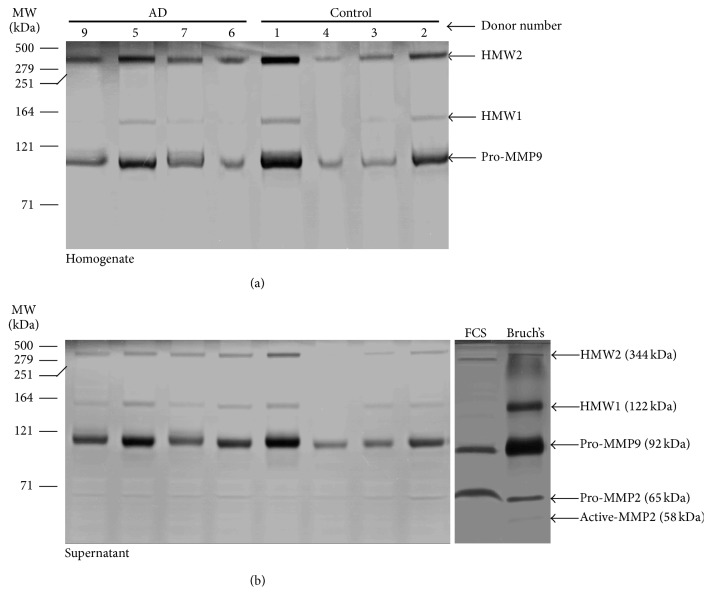
Components of the MMP Pathway in brain tissue. The MMP content of brain homogenates is shown in Gel A and the resulting profiles following centrifugation to obtain the supernatant fraction in Gel B. Altogether, 4 control and 4 AD brain samples were examined. HMW2 and pro-MMP9 were the predominant MMP species present in the homogenates with lower levels of HMW1. Pro-MMP2 was below the detection limit. Centrifugation to remove insoluble and membranous material resulting in the supernatant fraction showed a marked reduction in the level of HMW2 signifying that this species exists largely in the bound fraction. Supernatant fractions also demonstrated the presence of pro-MMP2. The molecular weights of MMP species present in brain were similar to those encountered in Bruch's membrane of the eye. Densitometry was undertaken to calculate the percentage of bound MMP species using (1) in the manuscript. The gels demonstrate the presence of the MMP Pathway in brain tissue.

A zymographic analysis of MMP species present in homogenates and their respective supernatants obtained from four control and four AD donor brains is shown in Figure 2. Sample from AD donor 8 was lost due to accidental shattering of the homogenizer tube. The amount of protein applied per lane for homogenate and supernatant fractions was 67 ± 1 *μ*g and 44 ± 4 *μ*g, respectively. For comparative purposes, the MMPs normally present in extracts of Bruch's membrane and in foetal calf serum (FCS) are also shown. The high molecular weight components of the MMP Pathway (HMW1 and HMW2) together with pro-MMP9 were clearly identifiable in the homogenates (Figure 2(a)). Removal of the insoluble (i.e., the pellet) fraction from the homogenate to obtain the supernatant fraction showed considerable loss of the HMW2 signal indicative of this component being largely in the bound form (Figure 2(b)). Levels of pro-MMP2 were too low to be detected in the homogenate fractions but just discernible in the supernatant fractions. The molecular weights of the MMP species present in brain samples were similar to those determined in Bruch's membrane.

Zymographic gels of Figure 2 showed considerable variation in the levels of individual MMP species between the various donors examined. This variation was quantified by undertaking a densitometric analysis, with activity being expressed as densitometric gel band area per *μ*g protein (Table 2(a)). The only significant difference was the lowered activity of HMW1 in the homogenate fractions of AD donors suggestive of lowered binding (*p* < 0.05).

MMPs present in the supernatant fraction are generally considered to be “free” whereas those in the pellet fraction are considered to be bound. However, MMPs bound within the LMMC complex also exist in the supernatant fraction and these are released on sample preparation for zymography. Thus the MMP species present in the supernatant fraction (Figure 2(b)) represents both free and those normally bound within the LMMC species. For this reason, it is best to consider MMPs in the supernatant fraction as being in the mobile phase. A procedure to separate LMMC from free MMPs by gel exclusion chromatography is described later.

In order to obtain the amount of MMP species present in the bound and mobile compartments, it would have been ideal to quantify levels in the supernatant and pellet. However, attempts at solubilisation of pellet proteins resulted in protein precipitation in the gel wells together with poor resolution and distortion of the MMP bands (results not shown). An alternative strategy was adopted, utilizing MMP quantification in the homogenate and supernatant fractions, to calculate the percentage in the bound/mobile compartments.

In the eight brain samples used to obtain Figure 2, the percentage of total protein in the tissue sample that was present (after homogenization and centrifugation) in the supernatant fraction was calculated as 32 ± 3% (Mean ± SD). Therefore, for a given MMP species, if *x* is the gel band area per microgram protein in the supernatant and *y* is the gel band area per microgram protein in the homogenate, the percentage of the species bound in the pellet (i.e., excluding that in the LMMC) is given by

(1) $$\begin{array}{c} \%\;\;bound=\frac{\left(y-0.32x\right)}{y}\times 10\text{0}. \end{array}$$

Since the protein levels in the homogenate and supernatant samples applied to the gels were known and the values of *x* and *y* for a given MMP species could be calculated from a densitometric scan of the gels, it was possible to calculate the percentage bound (Table 2(b)). In all donor samples, the HMW2 species existed in predominantly the bound state. In control tissue, about half of the HMW1 and pro-MMP9 content was found in the bound fraction whereas in AD donors, this binding was significantly reduced (*p* < 0.05). Bound pro-MMP2 species was not observed in any of the samples.

**Figure 3:**
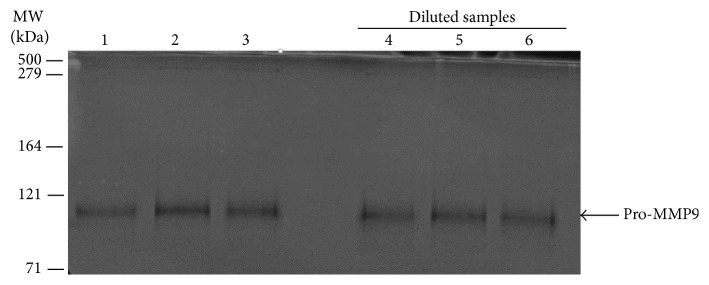
Effect of sample dilution on the release of bound MMPs. Supernatants from the undiluted and diluted brain homogenate (preparations A and B adjusted for dilution as described in the text) were subjected to zymographic analysis. Only the predominant pro-MMP9 species was detected but there was no significant difference in levels between preparations A and B. Lanes 1–3, preparation A. Lanes 4–6, preparation B.

In the above study, 50–100 mg of tissue was homogenized in 1.0 mL TBS and this variation in the amount of tissue used between donors may have altered the distribution of bound and free species, the degree being dependent on the magnitude of the dissociation constants. To assess the extent to which this may have influenced the binding data, tissue homogenate from donor number 2 was diluted tenfold and effects on the bound versus free level determined. To facilitate the comparison on the gels, supernatant from the concentrated homogenate was also diluted prior to zymography. If greater amounts of the bound form were released on dilution, then band intensities in the diluted samples should be higher. After a tenfold dilution of the homogenate, the levels of HMW2, HMW1, and pro-MMP2 were too low to be detected on the zymograms (Figure 3). Pro-MMP9, being present at much higher levels in tissues, was however detectable. There was no statistical difference in the level of pro-MMP9 present in the supernatants of undiluted and diluted samples (densitometric analysis of levels present in preparations A and B (see Materials and Methods) were 526 ± 39(3) and 497 ± 125(3) arbitrary units (mean ± SD(*n*)), respectively. The lack of increase in the level of pro-MMP9 in the supernatant of the diluted homogenate suggests that this MMP species remains tightly bound.

### 3.2. LMMC and Free MMPs in Brain Tissue

Supernatant extracts were obtained from four control and five AD donor samples and the protein content of each preparation was adjusted to 3.57 mg/0.8 mls. A 50 *μ*L aliquot was removed for zymography and the remainder fractionated by gel filtration chromatography on a Sepharose CL-6B column preequilibrated with TBS. The resulting fractions were then subjected to zymography.

**Figure 4:**
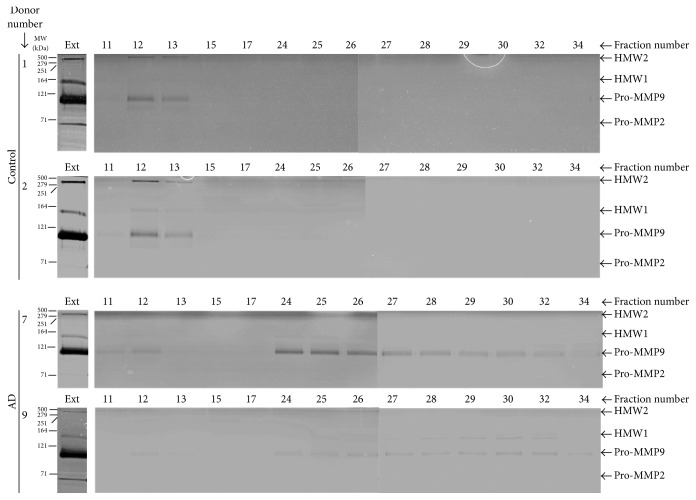
Presence of LMMC in brain tissue. Brain supernatant preparations (3.35 mg protein/0.75 mls) were fractionated on a gel filtration column (Sepharose CL-6B) and the resultant fractions were examined by zymography. Representative samples were obtained from two control (Gels 1 and 2) and two AD (Gels 7 and 9) donors. Gel lanes labelled Ext represent the content of MMP species present in the original supernatant extract applied to the column. The fractionation procedure resulted in considerable dilution of the MMP species. Due to its large size, the LMMC complex is expected to be appear at the elution forefront (fractions 11–13) followed by elution of the free MMP species (fractions 24 onwards). In control samples, LMMC constituents (HMW2 and pro-MMP9) are observed at the elution forefront but the gels show absence of free MMP species. In AD samples, MMPs associated with the LMMC complex are just discernible at the elution forefront but the gels are dominated by the presence of free MMP species.

A representative zymographic analysis of the fractions following elution is shown for two control and two AD donors in Figure 4. The gel lanes labelled “Ext” show the content of MMP species present in the original extract applied to the fractionation column. All these extracts were dominated by the presence of pro-MMP9 but pro-MMP2 levels were only discernible in donors 1 and 9. Elution of these extracts was associated with dilution of the MMP species such that band intensity in the corresponding fractions was considerably reduced.

The LMMC complex, due to its large size and exclusion from the column, is expected to be eluted at the forefront of the elution profile (fractions 11–13). Subsequent disintegration of the complex into its constituent MMP species during preparation for zymography results in the appearance of characteristic MMP species in the fractions at the elution front. Free MMP species, due to their low molecular weight, are expected to be eluted much later (fractions 24 onwards). In control samples (donors 1 and 2), HMW1, HMW2, and pro-MMP9 species were present at the elution front (signifying the presence of the LMMC complex) but free MMPs were not detected in the later fractions implying that most of the MMP species present in the supernatant was bound within the LMMC complex. In AD donor number 7, pro-MMP9 was discernible at the elution forefront showing the presence of LMMC but the vast majority of the species was present in the free form (eluting later in the fractionation profile). In AD donor 9 also, a trace of pro-MMP9 was present in the region associated with the LMMC complex (fraction 12) but the major signal was due to free HMW1 and pro-MMP9 eluting much later.

**Figure 5:**
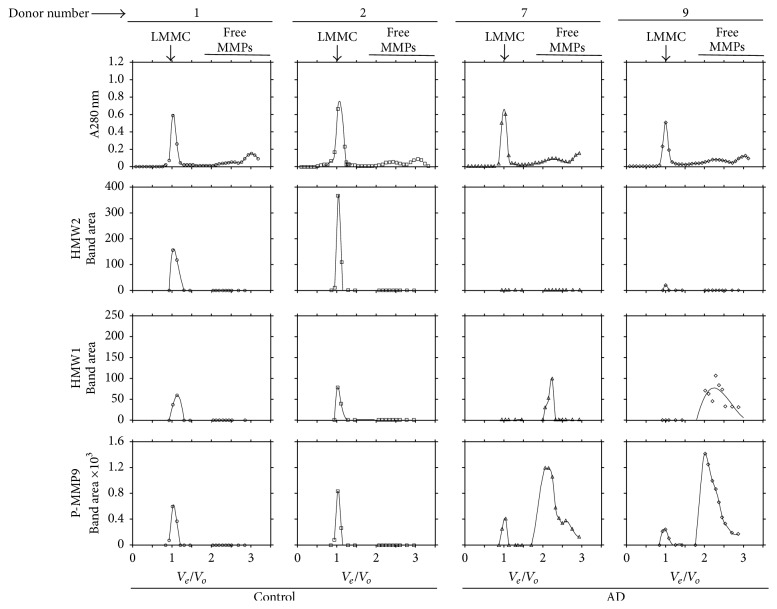
Densitometric quantification of MMP species following gel filtration and zymography. Scans were obtained from the zymograms in Figure 4 and are plotted as level of MMP species versus normalised elution volume (*v*_*e*_/*v*_*o*_). In the control plots, MMP species present were associated with the LMMC complex without any detectable amounts of free species. The AD plots were dominated by the copious presence of free HMW1 and pro-MMP9 species. Free HMW2 was not detected in any of the samples examined. Note the nearly 6-fold higher vertical scale in the pro-MMP9 plot compared to the HMW1 plot.

The photographic reproduction of the gels in Figure 4 suffers from lack of clarity and for quantitative purposes, densitometric scans were undertaken (Figure 5). Also included in Figure 5 are the absorption profiles of the fractions at 280 nm and these showed a predominant peak at the elution front. This not only reflects absorption by proteins but is largely due to light scatter by lipid-rich membranous aggregates appearing in the void volume which were too small to be removed by the centrifugation of the tissue extract. Figure 5 confirms the results from the gels in that control samples (donors 1 and 2) were dominated by the presence of LMMC with undetectable levels of free species. AD samples (donors 7 and 9) showed little or no HMW1 and HMW2 in the LMMC region but the presence of considerable free levels of HMW1 and pro-MMP9. It is unlikely that these differences between control and AD samples were due to postmortem instability of the LMMC complex for the following reason. Control donors 1 and 2 did not show detectable levels of free MMP species despite postmortem times of 39.5 and 32 hours. However, AD donor 7 with postmortem time of 14 hours showed the copious presence of both free HMW1 and pro-MMP9.

**Figure 6:**
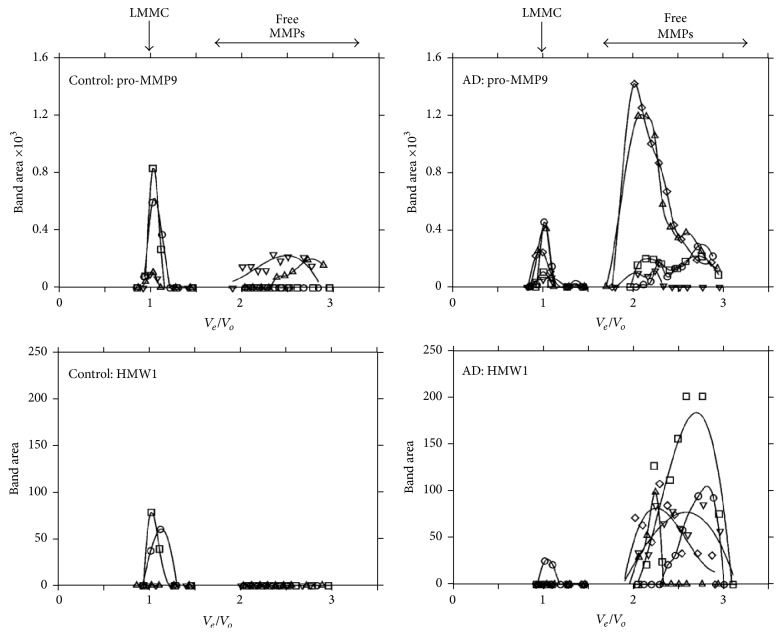
The relative distribution of HMW1 and pro-MMP9 in the LMMC complex and the free compartment. Data were obtained from the elution profiles of four control (numbers 1–4) and five AD (numbers 5–9) donors. In control donors, pro-MMP9 was clearly associated with the LMMC complex with some free pro-MMP9 in two of the donor samples. However the major portion of pro-MMP9 in AD samples existed in the free form (*p* < 0.05). HMW1 when present in control samples was complexed within the LMMC without any detectable free levels. In AD samples, the majority of the HMW1 species was present in free solution.

The elution profiles corresponding to pro-MMP9 and HMW1 species for all four control and five AD samples are depicted in Figure 6. In control samples, pro-MMP9 was present at the elution front indicative of the LMMC complex with some of the species present in the free form. AD samples also showed the presence of pro-MMP9 associated with the LMMC complex but the majority of the species existed in the free form. A quantitative assessment of free pro-MMP9 (by measuring the areas under the free MMP traces) showed significantly elevated levels in AD (control 93 ± 90_(4)_ mm^2^, AD 556 ± 440_(5)_ mm^2^, mean ± SD_(*n*)_, and *p* < 0.05).

In the supernatant, the HMW1 species, when present, was associated exclusively with the LMMC complex in control donors. In AD samples however, most of the HMW1 species was present in the free form.

## 4. Discussion

Gelatin zymography is an excellent technique for the identification of MMP activity associated with monomeric or covalently linked polymeric species but is prone to misinterpretation regarding physiological activity within the intact tissue. Pro-MMP2 and pro-MMP9, for example, are released into the extracellular compartment as inactive enzymes, but exposure to SDS in the zymographic buffers leads to conformational changes that expose the catalytic site making the enzyme partially “active.” Thus proteolytic activity is observed on the zymogram gels whereas physiologically, the enzymes are latent. Enzymatic activity on zymographic gels associated with pro-MMPs therefore only reflects potential substrate availability for activation. Active-MMPs are identified easily on zymographic gels due to their lower molecular weights (due to removal of the inhibitory peptide), running just below their parent proforms. However physiologically, these forms may also be inactive due to noncovalent binding of tissue inhibitors of MMPs (TIMPs). TIMPs are disassociated in the presence of SDS in the sample buffers for zymography. Therefore zymographic data requires cautious interpretation when extrapolating to the situation in intact tissue.

Analysis of intact brain tissue using techniques of in situ zymography or immunochemical localization cannot distinguish between latent and active forms of MMPs, between monomeric and polymeric forms, and does not give an indication of the presence of a bound-free equilibrium for specific species. Homogenization and subsequent electrophoretic separation of MMP species according to molecular weight (as part of the zymographic technique) allow identification and quantification of latent and active-MMP species and of high molecular weight complexes that are covalently linked. In the work of Backstrom et al., 1992 [48], brain hippocampal samples were disintegrated by vortex-mixing and sonication in the presence of SDS buffers and subsequent zymography showed the presence of four MMP species denoted as MP-70, MP-100, MP-130, and another MW higher than 205 kDa. These species were equivalent to the current designation from analysis of Bruch's membrane and other ocular tissues of pro-MMP2, pro-MMP9, HMW1, and HMW2, respectively [60]. There are two major drawbacks to tissue disruption in the presence of SDS buffers. Firstly, noncovalently bonded high molecular weight aggregates will also be solubilised; that is, the LMMC complex cannot be identified, and secondly, the bound/free equilibrium will be disrupted because SDS will also remove the bound species.

Homogenization in Tris buffers (in the absence of SDS) and centrifugation is expected to release intact the noncovalently bonded MMP complexes into the supernatant fraction for further analysis. It could be argued that complexes such as LMMC arise due to aggregation of free entities following the homogenization procedure. If this was the case, then higher levels of free MMP species should lead to higher levels in the LMMC complex. However, as shown in Figure 6, despite the much higher free level of pro-MMP9 and HMW1 in Alzheimer's samples compared to control, the amount present in the LMMC was much less than what would be expected if spontaneous aggregation was occurring.

Centrifugation of the homogenate to provide particulate and supernatant fractions allows an analysis of the bound-free distribution of a given MMP species. But this homogenization procedure could interfere with the bound-free equilibrium in that it may lead to dissociation of the species on dilution or the homogenization may expose hidden binding sites leading to greater sequestration. However as shown in Figure 3, pro-MMP9 was found to be tightly bound and even a 10-fold dilution did not disturb the bound-free equilibrium. More work is required to assess the dynamics of the equilibrium perhaps by determining the dissociation constants for individual MMP species.

The major gelatinase species associated with A*β* metabolism are MMP2 and MMP9 [61]. In most tissues (including brain), basal pro-MMP9 levels comprise the major MMP species and being “inducible,” pathophysiological signals arising from neovascular, inflammatory, or toxic metabolites such as A*β* result in greater expression of the latent form [33, 62–64]. Despite greater expression of pro-MMP9, these reported studies did not show increased active-MMP9. In the present study also, active-MMP9 was not observed on any of the gels and therefore the physiological importance for MMP9 involvement in A*β* metabolism must remain unresolved.

By contrast, pro-MMP2 is regarded as a constitutive enzyme with an increase in active levels in cultured neuronal cells on exposure to A*β* [45]. The upregulation of these MMPs in AD brain and in cell cultures exposed to A*β* suggests an important role for these enzymes in the disease process. The perplexing question remains as to why, despite the increased expression of these enzymes, levels of A*β* continue to accumulate. It is likely that the alternative route for removal of A*β*, namely, transport across the BBB, may also be compromised but the relative contribution of this pathway to the removal of A*β* is not known.

Increased expression does not necessarily translate to increased enzymatic activity because, as stated earlier, these pro-MMPs require the catalytic removal of a small inhibitory peptide for activation. Increased expression of pro-MMPs simply serves to elevate the amount of proenzyme available for the activation process. The actual amount available for activation is dependent on the relative contribution of secondary processes that can manipulate these levels by binding, cross-linking/polymerisation, and/or sequestration into higher molecular weight complexes. As explained earlier, in AMD, the >3-fold increase in free pro-MMP9 increased the complexation with pro-MMP2 to form HMW2 (via the MMP Pathway) resulting in decreased levels of active-MMP2 [23]. The operation of a similar pathway in brain could be informative regarding the free levels of pro-MMP2 and pro-MMP9, their potential for activation, and their likely contribution to degradation of A*β*.

In agreement with other studies, levels of pro-MMP9 in brain were always higher than levels of pro-MMP2 [33, 48, 49]. Quantitative estimations showed considerable variation in the level of MMP species between donors. In the case of pro-MMP9, for example, the standard deviations were between 33 and 74% of mean values in the various fractions analysed. Similar variation (51–91%) was observed in the analysis by Backstrom et al., 1992 [48] utilizing tissue from five control and seven AD donors. These authors using samples from the hippocampus also showed elevated levels of pro-MMP9 in AD, a finding not apparent in the present study. The reason for the discrepancy may be the high standard deviations, low donor numbers, or topographical variations since we examined parietal regions.

High molecular weight gelatinase species of 130 and 280 kDa (corresponding to the current designation of HMW1 and HMW2, resp.) had previously been noted in brain tissue but the relevance of these findings to control of free MMP levels was not investigated [48, 49, 65]. These studies also demonstrated elevated level of HMW1 in human AD [48] and elevated level of HMW2 in a canine model of AD [49].

The HMW2 species was virtually all bound in both control and AD brain samples. Of the total amount of HMW1 and pro-MMP9 present, about 50% was bound in controls but this binding was significantly reduced in AD (*p* < 0.05). Whether the “unbound” fraction (resident in the supernatant fraction) existed in a truly “free” form or in an equilibrium with LMMC was investigated using gel exclusion chromatography.

In control brain supernatant samples, the content of HMW1 and HMW2 was associated with the LMMC complex with the virtual absence of free forms. Pro-MMP9 was also associated with LMMC but some free pro-MMP9 was present in half of the control donors examined (Figure 6). Thus, despite the high content of HMW2 and pro-MMP9 in whole tissue, the free level of these species was very low. In AD brain samples, the amount of MMPs associated with LMMC was generally low indicative of lowered levels of this complex. Correspondingly, free HMW1 and pro-MMP9 species were present in all AD samples (Figure 6).

We need to consider the possibility that the LMMC complex may be an artefact arising from aggregation of MMP species with released cellular components during the homogenization procedure. In control tissue (Figures 5 and 6) there is very little free HMW1, HMW2, or pro-MMP9 and it could be argued that these species may be aggregating with released cellular components to form the LMMC complex. However this would seem unlikely since in AD samples, very high levels of free HMW1 and pro-MMP9 were present without a corresponding increase in the level of LMMC. LMMC is also present in Bruch's membrane that is essentially an extracellular matrix without cellular components. Further work is required to isolate the LMMC complex and define its protein and lipid content.

In summary, the MMP Pathway has been shown to be present in brain tissue with the potential to modulate the free levels of MMPs that can degrade *β*-amyloid. In Alzheimer's, preliminary results suggest dysregulation of the MMP system such that elevated levels of pro-MMP9 are predicted to compromise activation of MMP2 and thereby reduce the degradation of *β*-amyloid. Further work is now required, with a much larger donor group, to quantify the levels of the MMP2 species, and to evaluate the extent to which dysregulation of the MMP system affects the degradation potential towards *β*-amyloid in Alzheimer's disease.

## Figures and Tables

**Table 1:** Details of control and AD donors utilized in the study.

Donor number	Status	Age (years)	Sex	Histological diagnosis	Postmortemdelay (hours)	Braak stage
1	Control	74	F	No significant abnormalities	39.5	1
2	Control	86	F	No AD, moderate CAA	32	2
3	Control	85	M	No significant abnormalities	30.5	2
4	Control	79	F	No significant neuropathological abnormality	48	2
5	AD	78	M	AD moderate to severe	49.5	6
6	AD	81	M	AD probable	11	5
7	AD	85	F	AD definite, moderate atherosclerotic small vessel disease, moderate to marked CAA	14	5
8	AD	73	F	AD definite, moderate CAA	50.5	5
9	AD	78	F	AD definite	61	6

AD, Alzheimer's disease; CAA, cerebral amyloid angiopathy.

**Table:** (a) Gelatinase bands in the zymograms of Figure 2 were quantified by densitometry and activity expressed as gel band area per *µ*g protein (4 controls and 4 AD donors). Level of HMW1 in the homogenate of AD samples showed a significant reduction compared to controls. AD, Alzheimer's disease; NDet, not detected; ^*∗*^*p* < 0.05.

MMP species	MMP activity (gel band area/*μ*g protein) mean ± SD			
	Homogenate		Supernatant	
	Control	AD	Control	AD
HMW2	35.9 ± 24.2	35.5 ± 7.7	14.7 ± 13.2	10.4 ± 1.8
HMW1	6.4 ± 3.8	1.8 ± 2.0^*∗*^	8.3 ± 3.2	6.9 ± 5.0
Pro-MMP9	65.7 ± 48.6	51.8 ± 20	108 ± 75	111 ± 37
Pro-MMP2	NDet	NDet	2.33 ± 1.2	2.7 ± 0.4

**Table:** (b) For each MMP species in Figure 2, the gel band area per *µ*g of protein was determined for both homogenates and supernatants and the percentage of species bound was calculated using (1). HMW2 was observed to be tightly bound to the membranous or insoluble extracellular component in brain tissue. The amount of HMW1 and pro-MMP9 bound was significantly reduced in AD. Control donors: numbers 1–4; AD donors: numbers 5–7, 9. ^*∗*^*p* < 0.05.

MMP species	Percentage of bound MMP species (mean ± SD)	
	Control	AD
HMW2	93.8 ± 5.0	90.3 ± 1.3
HMW1	47.3 ± 24	13.3 ± 14.3^*∗*^
Pro-MMP9	55.5 ± 11.8	28.5 ± 15.4^*∗*^
Pro-MMP2	0	0
